# Courage to Cobble Something New: Creative Representations of Bisexuality and Aging

**DOI:** 10.1093/geroni/igaa057.2068

**Published:** 2020-12-16

**Authors:** Sarah Jen, Rebecca Jones

**Affiliations:** 1 University of Kansas, Lawrence, Kansas, United States; 2 Open University, Milton Keynes, England, United Kingdom

## Abstract

There are few cultural representations or scripts available for LGBTQ aging. Among bisexual and otherwise non-monosexual (bi+) women, stereotypes of hypersexuality exclude older adults while the contrasting experience of invisibility obscures the existence of bi+ aging. In this discourse analysis, we examined three issues of the Bi Women Quarterly (BWQ) newsletter published between 2014-2019 which were devoted to the intersection of aging and bisexuality. Data include 42 narratives, personal reflections, interviews, poems, letters, advice columns, and photos which were analyzed to identify linguistic tools, visual imagery, and broader discourses used to construct and convey the meaning and experience of bisexual aging. Themes include: 1) lacking a “blueprint” for bisexual lives, 2) significant “turning points,” 3) intergenerational (dis)connections across history, and 4) life-long patterns of discovery and disclosure. More visible and diverse narratives for bisexual aging might better enable bi+ individuals to envision and effectively plan for their own aging futures.

